# Percutaneous full-endoscopic uniportal decompression for the treatment of symptomatic idiopathic lumbar spinal epidural lipomatosis: Technical note

**DOI:** 10.3389/fsurg.2022.894662

**Published:** 2022-09-06

**Authors:** Yong Yu, Ye Jiang, Fulin Xu, Lutao Yuan, Yuhang Mao, Chen Li

**Affiliations:** ^1^ Department of Neurosurgery, Zhongshan Hospital, Fudan University, Shanghai, China; ^2^ Department of Neurosurgery, Minhang Hospital, Fudan University, Shanghai, China

**Keywords:** spinal epidural lipomatosis, percutaneous, minimally invasive surgery, uniportal, full-endoscopic

## Abstract

**Background:**

Lumbar spinal epidural lipomatosis (SEL) is a rare condition characterized by an excessive accumulation of adipose tissue within the spinal canal, compressing the dura sac and/or nerve roots. When conservative treatments fail and clinical symptoms progress quickly and seriously, surgical decompression should be considered. With the rapid development of endoscopic armamentaria and techniques, the pathological scope that can be treated by percutaneous endoscopic spine surgery is ever expanding.

**Objective:**

In this paper, the authors describe a patient with lumbar spinal epidural lipomatosis who was treated with a percutaneous full-endoscopic uniportal decompression surgery successfully. This article aims to validate the feasibility of percutaneous full-endoscopic uniportal decompression for the treatment of symptomatic idiopathic spinal epidural lipomatosis *via* interlaminar approach.

**Methods:**

We describe a case of a 69-year-old man with a 10-year history of low back pain, intermittent claudication, and bilateral leg neuropathic pain. He was diagnosed with lumbar epidural lipomatosis, which did not respond to conservative therapy. After a comprehensive evaluation, he underwent percutaneous endoscopic spine surgery to remove hyperplastic adipose tissue and decompress nerve roots and dura sac.

**Results:**

The patient was treated with a percutaneous full-endoscopic uniportal decompression surgery successfully. After the procedure, his leg pain decreased and his walking capacity improved. There were no surgery-related complications, such as cerebrospinal fluid leakage, incision infection, etc.

**Conclusions:**

The case with SEL was successfully treated with a percutaneous full-endoscopic uniportal surgery, which has the advantages of excellent presentation of anatomical structures, expanded field of vision, less surgical-related trauma, and bleeding. The key point of the procedure is to release and cut off the bands which divide the epidural space into small rooms filled with excess adipose tissue.

## Introduction

Percutaneous endoscopic spine surgery has been evolving rapidly these years with the development of endoscopic philosophy, technology, and equipment (1, 2). Consequently, the indications of endoscopic spine surgery are ever expanding, from the initial lumbar intervertebral disk disease to other types of pathologies located in the whole spinal column (3, 4). The obvious advantages of working-channel endoscopic spinal surgery include the reduction of the surgical corridor, avoiding soft tissue and muscular stripping, minimizing bony resection, as well as obtaining excellent visualization (1, 2, 4).

**Figure 1:**
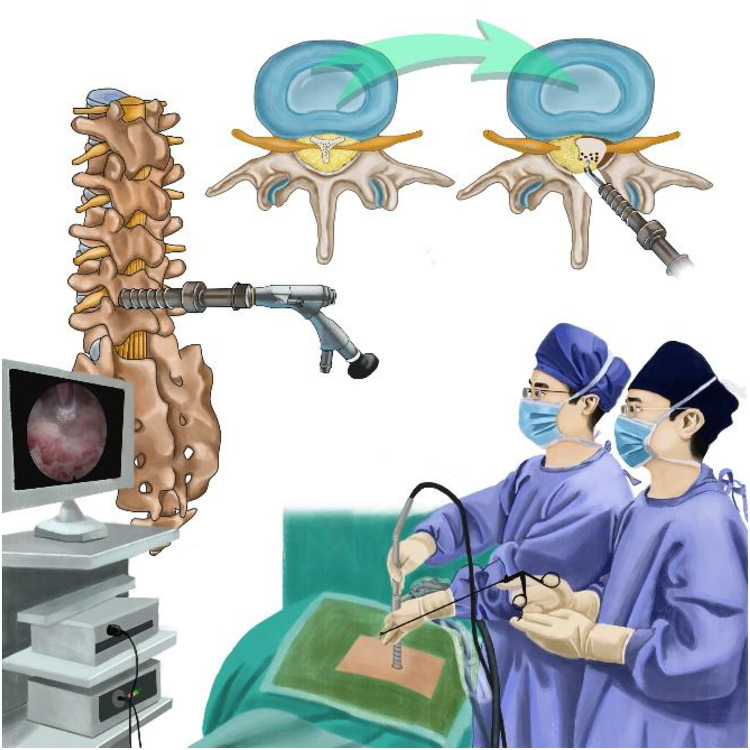
Illustration showing the percutaneous full-endoscopic uniportal decompression for the treatment of spinal epidural lipomatosis.

Since Lee et al. first reported a case of spinal epidural lipomatosis (SEL) in 1975 (5), more and more studies on this disease have been retrieved in the literature (6–12). SEL is defined as an abnormal accumulation of adipose tissue in the epidural space within the spinal canal resulting in compression to the spinal cord and/or cauda equina. Clinical manifestations of SEL in lumbar include low back pain, lower extremity weakness, lower extremity numbness, and neurogenic intermittent claudication, which are identical to that of degenerative lumbar stenosis. The treatment measures of SEL include conservative therapy and surgical decompression. Although there is still no clear consensus on the treatment of SEL, the approach to patients with SEL should initially be conservative involving weight reduction and endocrine therapy (13, 14). Surgery interventions should be considered when conservative treatments fail and clinical symptoms deteriorate rapidly (14). As for the surgical methods, extensive laminectomy and excision of the adipose tissue are the most commonly used options (15). So far, there has been no report to treat lumbar lipomatosis using the percutaneous uniportal full-endoscopic technique. In this report, we describe a case of lumbar epidural lipomatosis, which was successfully treated with percutaneous uniportal full-endoscopic surgery (Figure 1). The objective of this article is to validate the feasibility of the approach and describe several operative pearls based on our experience.

## Materials and methods

The study was conducted in accordance with the guidelines of the 1964 Declaration of Helsinki and was approved by the ethics committee of Zhongshan Hospital, Fudan University (Institutional Review Board approval number 2021-042), as well as Minhang Hospital, Fudan University (Institutional Review Board approval number 2021-037-01X). The patient signed informed consent forms for the surgery procedure.

### History and examination

A 69-year-old male patient (weight, 70 kg; height, 172 cm; body mass index, 23.66 kg/m^2^) presented with a 10-year history of low back pain, neurogenic intermittent claudication, and bilateral leg radicular pain. He could walk no more than 100 m and daily activities were severely affected. His VAS score for leg pain was 8/10. He denied any history of endocrine and metabolic diseases and steroid use. Physical and neurologic examinations showed that the dorsiflexor strength of bilateral ankles and great toes was grade 4. Besides, the physical examination also indicated that there was numbness accompanied by a decrease in temperature, touch, and pinprick sensation in the skin of the bilateral sole and calf. His numbness, pain, and walking capacity did not respond to conservative treatment measures, including physical therapy, weight control, and oral medications. He did not receive an epidural steroid injection.

**Figure 2:**
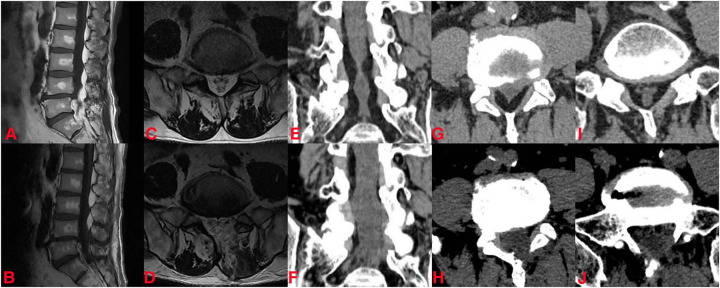
The preoperative and postoperative imaging data of the patient. Preoperative (**A**) and postoperative (**B**) sagittal MRI image; preoperative (**C**) and postoperative (**D**) axial magnetic resonance imaging (MRI) image at the L5–S1 level; preoperative (**E**) and postoperative (**F**) coronal CT; preoperative (**G**) and postoperative (**H**) axial CT at L4–5 level; preoperative (**I**) and postoperative (**J**) axial CT at the L5–S1 level.

His lumbar dynamic x—ray radiographs did not show any instability. Lumbar magnetic resonance imaging (MRI) and computed tomography (CT) reconstruction revealed abnormal deposition of epidural adipose tissue in the spinal canal, which compressed the nerve roots and thecal sac, especially at the L4–5 and L5–S1 levels (Figure 2). He was diagnosed with idiopathic lumbar spinal epidural lipomatosis. In accordance with the MRI grading by Borré et al. (16), the current state of the patient was classified as grade III.

### Endoscopic instruments

**Figure 3:**
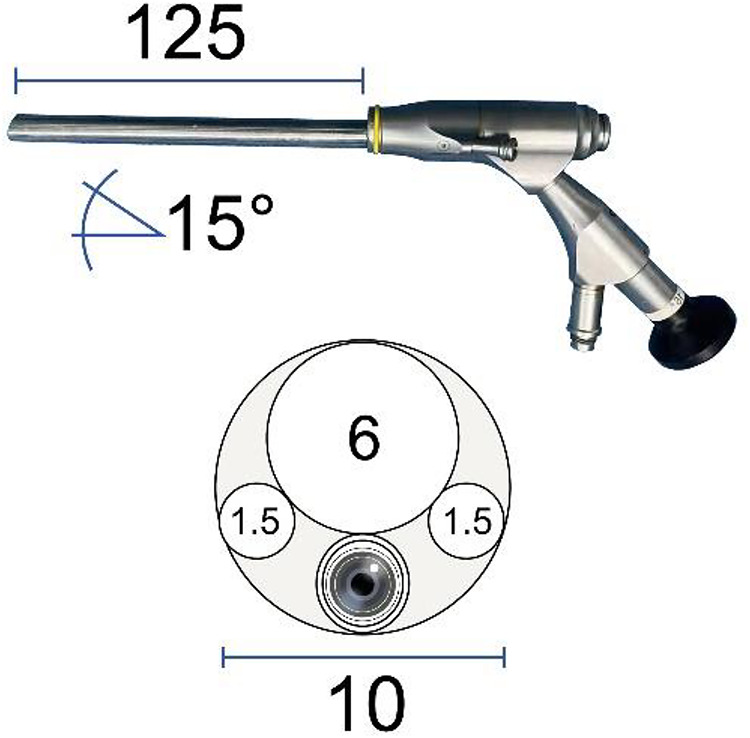
Illustration showing endoscopic surgical system Delta (Joimax GmbH, Karlsruhe, Germany) scope profile, including outer diameter, working channel, working length, and angle.

The endoscopic surgical system Delta (Figure 3) (Joimax GmbH, Karlsruhe, Germany) was applied to perform the surgery, including an endoscope (15° angle), endoscopic sheaths, basket forceps, endoscopic punches, nucleus pulposus clamp, etc. The radiofrequency probe (Trigger-FlexR Bipolar System, Elliquence LLC, Baldwin, NY, USA) was used to ablate soft tissue and control bleeding. The endoscopic high-speed diamond burr (Primado P200-RA330, NSK-Nakanishi International, Co., Ltd., Osaka, Japan) was utilized to grind bones.

### Operative technique

**Figure 4:**
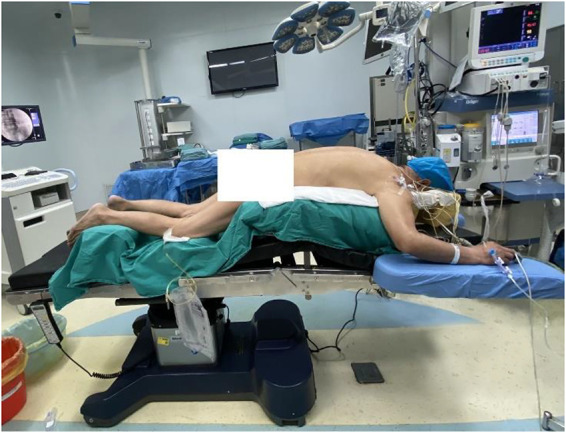
The patient was positioned in the prone position on a radiolucent surgery table with hips and knees in slight flexion and hands appropriately oriented at the sides of the head.

The operation was performed under general anesthesia. The patient was placed in a prone position on a radiolucent surgery table with appropriate flexion (Figure 4). The posterior approach was used to perform the decompression and debulk at L4–5 and L5–S1 levels.

**Figure 5:**
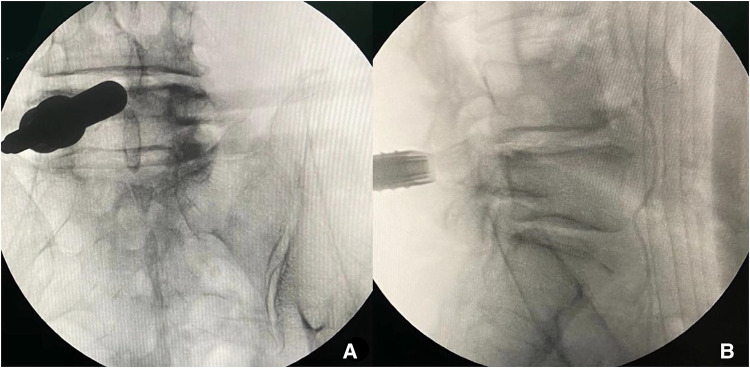
The position of the working cannula was verified with fluoroscopy in anteroposterior and lateral position (L4–5).

Lumber 4–5 segment was performed firstly. After the patient was routinely sterilized and draped. A 10 mm stab wound was made in the skin and a pencil-like rod was introduced to touch the bone (left L4–5 articular) under fluoroscopic guidance. The paravertebral muscles and fascia were dilated gradually by soft-tissue-dilators. Then, the 10 mm delta working cannula with oblique mouth was inserted. The position of the working cannula was verified with fluoroscopy in anteroposterior and lateral positions (Figure 5A, B). Finally, the endoscopic surgical system was introduced and all the subsequent steps were performed under constant irrigation with endoscopic visualization.

**Figure 6:**
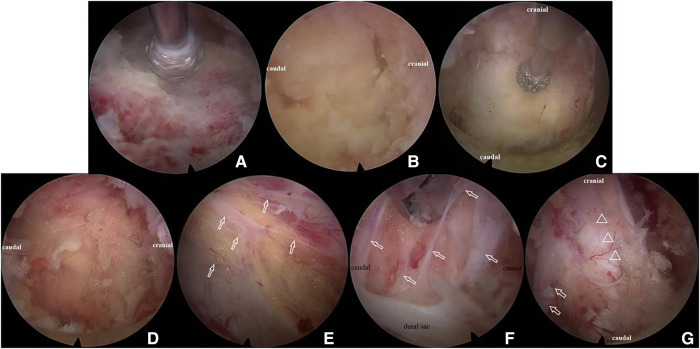
Illustration of percutaneous full-endoscopic uniportal decompression procedures for the treatment of lumbar spinal epidural lipomatosis. (**A**) The inter lamina window was expanded using diamond bur; (**B**) the cranial and caudal end of the ligamentum flavum was identified; (**C**) the base of the spinous process was shaved with diamond bur without removal of the ligamentum flavum; (**D**) epidural excess fatty tissues were exposed; (**E**) bands (white arrow) between dural sac and ligamentum flavum; (**F**) bands (white arrow) between the dura mater and the nerve root; (**G**) decompression of the dural sac (triangle) and bilateral traversing nerve root (white arrow).

After the soft tissue was cleared using the radiofrequency probe, the left L4 lamina, L5 lamina, and ligamentum flavum between L4 and L5 could be identified under endoscopy. The ipsilateral partial laminotomy (L4 lower part and L5 upper part), as well as partial facet joint resection, were performed using a 3.5 mm endoscopic diamond bur and endoscopic Kerrison Rongeur (Figure 6A). The outer layer of ligamentum flavum was removed with a rongeur to expose the inner layer, which was tightly attached to the inner surface of the lamina, especially on the cephalic side (Figure 6B). The inner layer of ligamentum flavum was retained *in situ* in this step to prevent bleeding. Then, the base of the spinous process was shaved to facilitate that the working cannula can be inserted toward the contralateral side (Figure 6C). Contralateral bony decompression could be achieved using a diamond bur between the undersurface of the lamina and the ligamentum flavum (17). The enthesis of the inner ligament was detached and at last, the whole ligamentum was resected. At the same time, epidural excess fatty tissues were identified (Figure 6D). It should be noted that abundant bands were found not only between the dura sac and inner ligament, but also between the dura mater and the nerve root (Figures 6E,F). These bands divided the epidural space into small rooms filled with excess fat. The bands were cut off and most of the fatty tissues were removed. At the end of the operation, decompression of bilateral traversing nerve roots, as well as pulsation of the thecal sac were confirmed (Figure 6G). The skin incision was closed with one stitch. The same surgical technique was used for the adjacent affected level (L5–S1 level).

## Results

After the procedure, his leg pain decreased and his walking capacity improved. One day postoperatively, the pain decreased sufficiently with 2/10 on VAS. There were no surgery-related complications, such as cerebrospinal fluid leakage, incision infection, etc. During the follow-up, 3- and 12-month postoperatively, the VAS scores were 2/10 and 1/10 points. He was able to walk 500 m without obvious numbness and pain in his lower limbs at the last follow-up. Postoperative MRI and CT scans indicated the successful decompression of the neural structure at the L4–5 and L5–S1 levels (Figure 2).

## Discussion

Under physiologic conditions, the epidural fat tissue in the spine canna is thought to serve as a cushion for nerve structures. However, the excessive accumulation of fat tissue, which is referred to as SEL, can cause compression damage to the spinal cord and cauda equina. As a result, patients with SEL often develop neurological symptoms, such as sensory and motor disturbance, claudication, lower back pain, and radiculopathy (14). The pathogenesis of SEL may include the following disease states: endogenous steroid hormonal disease, long periods of exogenous steroid use, surgery-induced, obesity, and idiopathic disease (18, 19). Malone et al. reported a rate of 6.26% for symptomatic SEL in their population, with an incidence rate of 2.5% per year (9). Thus, it can be seen that SEL seems to be more than people originally thought. The patient we presented in this report had difficulty in walking less than 50 m, leg weakness, and sensory loss in L4, L5, and S1 distribution. He had no history of endocrine diseases and steroid use. Therefore, he was diagnosed with idiopathic SEL.

For this idiopathic SEL patient, with clinical symptoms have been deteriorating progressively, surgical treatment should be considered (14). Although there is no consensus about surgical methods, laminectomy with excision of the hypertrophic fatty tissues is considered the mainstay (6, 12, 14, 20, 21). However, the traditional laminectomy and microsurgery of excision of the hypertrophic fatty tissue need a wide incision, significant paravertebral muscle stripped, and bone dissection for adequate visualization, which can result in postoperative pain and slow recovery (22). During the past three decades, percutaneous endoscopic spine surgery has evolved dramatically with the development of endoscopic equipment and techniques. As a result, the indications of endoscopic spine surgery are ever expanding, from the initial lumbar disc disease to other types of pathologies located in the whole spine column (4). The obvious advantages of percutaneous working-channel endoscopic spine surgery are as follows: reduction of the surgical corridor, avoiding muscular dissection, reduction of bony resection to prevent iatrogenic instability, close observation to obtain excellent visualization, and reduction of bleeding under water perfusion pressure (4). In the operation of this case, we used Delta endoscopic surgical system with a larger manipulation channel (diameter of 1 cm) than the previous traditional endoscopy (Figure 3). This character facilitated us to use larger sizes of endoscopic Kerrison punches in the procedure, in which the size of the rongeur bite part can be up to 5 mm. Hence, the ligamentum flavum and its enthesis are convenient to be removed (17). Owing to this, the efficiency of surgery was greatly enhanced. The operation duration was within 2 h for two segments (L4–5, L5–S1) in this case. Kang et al. reported that the biportal endoscopic technique had been used to achieve successful neural decompression for symptomatic SEL (22). It is indeed a minimally invasive technique possessing the traits of percutaneous spinal endoscopy, such as close observation and clear field of vision under water irrigation. Compared with this technique, our uniportal technique had less damage to soft tissue because we neither need two skin incisions and two ports nor need to remove part of the muscle and muscle fascia to make room for the procedure and continuous irrigation. In this case, our experience has proved that the full endoscopic uniportal technique is fully suitable for decompression treatment for SEL.

In addition, compared with microscopic channel surgery, our endoscopic surgery has great strengths to manipulate the opposite side lesion. The outer working cannula can be inserted toward the contralateral side using sub-spinous process space. The camera’s eye with 15 view angles can be put closer to the opposite side lesion, providing high-definition images on a video monitor for the operator (4, 22). Therefore, it makes the removal of excessive adipose tissue safer and easier, facilitating the reduction of the possibility of dura tear and neural damage.

**Figure 7:**
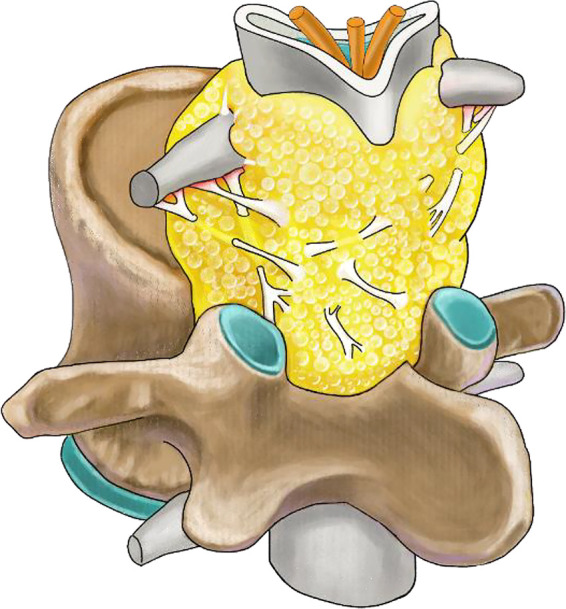
Illustration showing mechanisms of compression of the nerve roots and dura sac in spinal epidural lipomatosis.

Thanks to the high-definition vision of endoscopy, we had an interesting discovery during the operation. We found that there were many bands among the dura, ligamentum flavum, and nerve roots in the epidural space (Figures 6E,F). These bands separated the epidural space into multitudinous small spaces, and the hyperplastic adipose tissue was bound in these small spaces. It can explain why although adipose tissue is soft, it causes compression of the nerve roots and dura sac (Figure 7). In this case, the thecal sac has a striking stellate appearance on lumbar axial imaging. Kuhn et al. named this configuration as the “Y-sign” which is characteristic of lumbar SEL (23). The stretch action of these bands may interpret the “Y-sign.” During the operation, cutting off these bands to eliminate the stretch action is very important to release the compression of the sac and nerve roots. Cutting off these bands also makes it easier to remove fatty tissues and reduces bleeding and dura involvement. Frank reported an endoscopic suction technique for the treatment of idiopathic epidural lipomatosis (24). In his article, he noted that sharp microsurgical techniques should be used in the area that the fat was well vascularized and adherent to the dura. Otherwise, suction of adipose tissue is insufficient to achieve success. Our experience verified that in current uniportal full-endoscopy technology, all the procedures can be accomplished in a single channel.

### Limitations

To our knowledge, we reported the first use of percutaneous uniportal full-endoscopic decompression for the treatment of lumbar SEL disease. The limitation of this article is obvious, which is a case report without enough follow-up time. However, this article aims to validate the feasibility of percutaneous full-endoscopic uniportal decompression for the treatment of symptomatic idiopathic spinal epidural lipomatosis. We believe that it can be used as a reference for other doctors who are going to employ this technique.

## Conclusion

The case with SEL was successfully treated with a percutaneous full-endoscopic uniportal surgery, which has the advantages of excellent presentation of anatomical structures, expanded field of vision, less surgical-related trauma, and bleeding. The key point of the procedure is to release and cut off the bands which divide the epidural space into small rooms filled with excess adipose tissue.

## Data Availability

The original contributions presented in the study are included in the article/Supplementary Material, further inquiries can be directed to the corresponding author/s.
